# The complete chloroplast genome sequence of *Mentha canadensis* (Labiatae), a traditional Chinese herbal medicine

**DOI:** 10.1080/23802359.2019.1687031

**Published:** 2019-12-09

**Authors:** Li Huaizhu, Yang Lin, Jiqing Bai, An Qiuyan, Dou Lingling, She Rui-Xue

**Affiliations:** a School of Chemistry and Chemical Engineering, Xianyang Normal University, Xianyang City, China;; b Shaanxi University of Chinese Medicine, Xianyang City, China;; c Key Laboratory of Resource Biology and Biotechnology in Western China, Ministry of Education, College of Life Sciences, Northwest University, Xi’an, China

**Keywords:** *Mentha canadensis*, chloroplast genome, Labiatae

## Abstract

*Mentha canadensis* is a well-known resource of traditional Chinese herbal medicine, belonging to the *Mentha* of the Labiatae family. In this study, the whole chloroplast genome of the *M. canadensis* chloroplast genome was sequenced, assembled and annotated, which contains 134 unique genes, including 89 protein-coding genes, 37 tRNA genes and 8 rRNA genes. A maximum likelihood phylogenetic tree based on 11 complete chloroplast genomes revealed that *M. canadensis* is closely related to *M. longifolia* and *M. spicata*. The chloroplast genome could be used for variety identification, genetic engineering and effective protection of germplasm resources.

The *Mentha canadensis* is a species of genus *Mentha* and mainly distributed within 0–3500 m of wet areas in Asia and North America. It is widely cultivated for its essential oils, and also used as raw materials in Chinese herbal medicine, food, cosmetics, and other industries (Jirovetz et al. 2009). To date (9/15/2019), more than 30 different Labiatae species chloroplast genome have been sequenced and deposited into the National Center for Biotechnology Information (NCBI). However, the chloroplast genome of the *M. canadensis* has not been reported. Here, we first reported the complete chloroplast genomes of *M. canadensis* based on Illumina Hiseq pair-end sequencing data.

The sample of *M. canadensis* was collected from Jiaokou town, Yanchang county in the Shaanxi Province (Geographic coordinates: 36°28′33.5″N, 110°5′10.7″E; Altitude: 884.3 m). The voucher specimen of *M. canadensis* is kept at the herbarium of Shaanxi University of Chinese Medicine (Xianyang, Shaanxi, China), accession number: 610621180727003LY. The total genomic DNA was extracted using a modified CTAB method (Stefanova et al. 2013). Total reads were mapped to reference (*M. longifolia* chloroplast genome: NC_032054) and the mapped reads were extracted and assembled by MIRA (Chevreux et al. 2004). The assembled chloroplast genome was annotated and corrected using Geneious (Kearse et al. 2012). The complete chloroplast genome sequence of *M. canadensis* was deposited into GenBank (accession number MN047448).

The complete chloroplast genome of *M. canadensis* is 152,154 bp in length, containing two inverted repeats (IRa and IRb: 25,600 bp), a large single copy (LSC: 83,278 bp), and a small single copy (SSC: 17,676 bp). A total of 134 genes were annotated, including 89 protein-coding genes, 37 tRNA genes, and 8 rRNA genes. The GC content of the complete chloroplast genome and protein coding region (CDS) are 37.8% and 38.1%, respectively.

The maximum-likelihood phylogenetic tree was generated based on the complete chloroplast genome of *M. canadensis* and eight other Labiatae species (Figure 1). The results showed that *M. canadensis* was closely related to the same genus species *M. longifolia* and *M. spicata.* Sequencing of the complete chloroplast genome of *M. canadensis* would lay foundations for variety identification, genetic engineering, and effective protection of *Labiate* germplasm resources.

**Figure 1.**
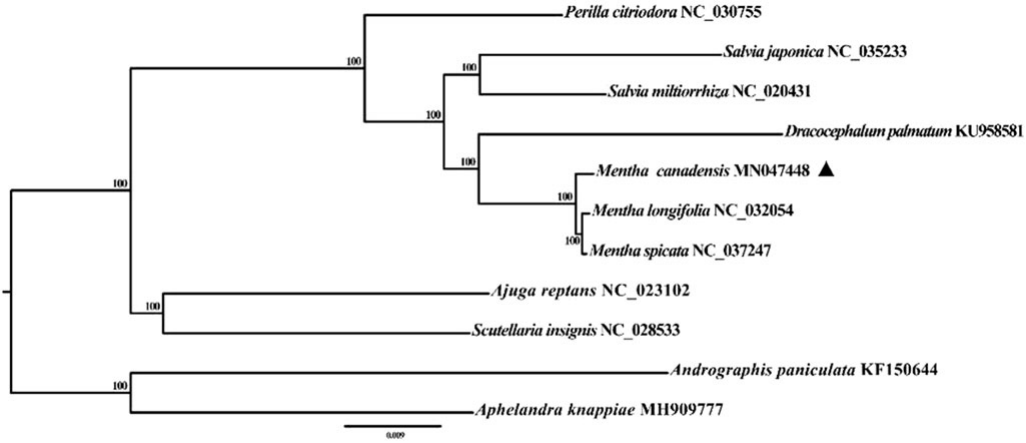
Maximum-likelihood phylogenetic tree base on 11 completely chloroplast genome. The accession numbers are shown in the figure. Bootstrap support values based on 1000 replicates are displayed on each node. Marked by a black triangle is *M. canadensi* in this study.
